# Smoking Cessation Intervention on Facebook: Which Content Generates the Best Engagement?

**DOI:** 10.2196/jmir.4575

**Published:** 2015-11-11

**Authors:** Johannes Thrul, Alexandra B Klein, Danielle E Ramo

**Affiliations:** ^1^ Center for Tobacco Control Research and Education University of California, San Francisco San Francisco, CA United States; ^2^ Department of Psychiatry University of California, San Francisco San Francisco, CA United States

**Keywords:** young adults, smoking cessation, Facebook, engagement, Transtheoretical Model

## Abstract

**Background:**

Social media offer a great opportunity to deliver smoking cessation treatment to young adults, but previous online and social media interventions targeting health behavior change have struggled with low participant engagement. We examined engagement generated by content based on the Transtheoretical Model of Behavior Change (TTM) in a motivationally tailored smoking cessation intervention on Facebook.

**Objective:**

This study aimed to identify which intervention content based on the TTM (Decisional Balance and 10 processes of change) generated the highest engagement among participants in pre-action stages of change (Precontemplation, Contemplation, and Preparation).

**Methods:**

Participants (N=79, 20% female, mean age 20.8) were assessed for readiness to quit smoking and assigned to one of 7 secret Facebook groups tailored to their stage of change. Daily postings to the groups based on TTM Decisional Balance and the 10 processes of change were made by research staff over 3 months. Engagement was operationalized as the number of participant comments to each post. TTM content-based predictors of number of comments were analyzed and stratified by baseline stage of change, using negative binomial regression analyses with and without zero inflation.

**Results:**

A total of 512 TTM-based posts generated 630 individual comments. In Precontemplation and Contemplation groups, Decisional Balance posts generated above average engagement (*P*=.01 and *P*<.001). In Contemplation groups, posts based on the TTM processes Dramatic Relief and Self-Liberation resulted in below average engagement (*P*=.01 and *P*=.005). In Preparation groups, posts based on Consciousness Raising generated above average engagement (*P*=.009). Participant engagement decreased over time and differed between groups within Precontemplation and Contemplation stages, but was independent of day of the week and time of day the content was posted to the groups. No participant baseline characteristics significantly predicted engagement.

**Conclusions:**

Participants not ready to quit in the next 30 days (in Precontemplation or Contemplation) engaged most when prompted to think about the pros and cons of behavior change, while those in the Preparation stage engaged most when posts increased awareness about smoking and smoking cessation. Findings support tailoring intervention content to readiness to quit and suggest intervention components that may be most effective in generating high participant engagement on social media.

## Introduction

Smoking is the biggest behavioral risk factor for premature morbidity and mortality in the United States, and young adults are the age group with the highest smoking prevalence [1]. Young adults are also an underserved population given that few are reached by currently available evidence-based smoking cessation interventions [2,3]. Novel cessation interventions to reach young adults smokers are clearly needed.

Recent reviews of Internet [4] or social media interventions [5] for health behavior change and a meta-analysis of Web- and computer-based smoking cessation interventions [6] have concluded that these kinds of interventions can have small but significant beneficial effects (eg, effects of Web-based interventions on smoking cessation—relative risk 1.40) [6]. However, low participant engagement with online or social media interventions is a critical obstacle to improving health behavior outcomes [5,7,8]. Research on Internet-based health promotion interventions suggests that peer and counselor support, email/telephone contact, and regular updates to the website improve participant engagement [9]. Further, the results of a Web-based smoking cessation intervention suggested that a prescriptive message tone (vs a motivational tone), a dictated content viewing order (vs navigation autonomy), and email reminders increased engagement among participants [10]. Compared to other online interventions, social media offer several potential advantages for participant engagement, since they can reach large audiences [11] that are already regular users and familiar with the platform. Further, social media are typically more interactive and require the user to engage more than traditional websites [12] and can promote social connectedness and sharing of experiences [13]. However, little is known about how characteristics of social media interventions are related to participant engagement.

The Transtheoretical Model (TTM) is a well-researched theory of health behavior change [14,15], conceptualizing the process of behavior change into five different stages: Precontemplation (not ready to change in the near future), Contemplation (intending to change within the next 6 months), Preparation (intending to change within the next 30 days), Action (achievement of intended change for less than 6 months), and Maintenance (achievement of intended change for 6 months or more) [15]. Additional dimensions of the TTM include Decisional Balance, or the balance between pros and cons of the problematic behavior and of behavior change [16], and 10 processes of change that aid progression from one stage to the next [17]. See Table 1 for an overview and definition for all TTM content used in the current study.

**Table 1:** Transtheoretical Model (TTM) content (Decisional Balance and 10 processes of change).

TTM content	Definition
Decisional Balance	Pros and cons of behavior and behavior change
Counter-Conditioning	Substituting healthy alternative behaviors and thoughts for old behaviors
Consciousness Raising	Learning new facts, ideas, and tips that support the behavior change
Dramatic Relief	Experiencing negative emotions that go along with old behaviors and positive emotions that go along with new behaviors
Environmental Reevaluation	Realizing the negative impact of one’s behavior and the positive impact of change on others
Helping Relationships	Seeking and using social support to make and sustain change
Reinforcement Management	Increasing rewards for healthy behavior change and decreasing the rewards for old behaviors
Stimulus Control	Removing reminds/cues to engage in old behavior, and using cues to engage in the new healthy behavior
Self-Liberation	Making a firm commitment to change
Social Liberation	Realizing that social norms are changing to support new behavior
Self-Reevaluation	Realizing that the behavior change is an important part of one’s identity

Previous research suggests that processes of change focused on cognitions, affects, and evaluations are more appropriate in earlier stages, while commitment, conditioning, and stimulus control are more appropriate in the more advanced stages [18]. The TTM and its components are frequently used to design Internet-based interventions to promote health behavior change and TTM-based interventions were found to result in significant effects on health-related behaviors including smoking cessation [4,19,20]. With regard to smoking cessation interventions on Facebook, mixed-methods formative work suggested that an intervention tailored to readiness to quit smoking would likely appeal to the widest range of young adult smokers [21]. Yet, there is no evidence as to how social media intervention content should be tailored to best engage young adult smokers who are in different stages of change.

Engagement in Facebook interventions to change health behavior has previously been operationalized as posting, commenting, or liking of content [22,23], and previous studies also reported that engagement in social media interventions more generally tends to decrease over time [23-25]. In order to develop effective social media interventions for smoking cessation, it is important to know how intervention content should be tailored to produce high participant engagement. However, to our knowledge, no study has examined how intervention content is associated with participant engagement in a smoking cessation intervention delivered through Facebook. Intervention engagement is a meaningful outcome to study, since results from behavioral smoking cessation counseling studies suggest that abstinence increases with overall contact time (up to 90 minutes) as well as with number of treatment sessions [26]. With regard to digital health interventions, one previous study suggests that higher engagement in a Web-based smoking cessation intervention was positively associated with smoking cessation outcomes [27].

In the context of a feasibility trial of the Tobacco Status Project, a Facebook smoking cessation intervention for young adults, this study aimed to identify which intervention content based on the TTM generated the highest engagement among participants in pre-action stages of change (Precontemplation, Contemplation, Preparation).

## Methods

### Procedure

Recruitment efforts included a paid Facebook ad campaign conducted between June and August 2013 actively targeting young adults 18-25 years old, with details reported previously [28]. When meeting eligibility criteria and consenting to study participation, participants were assigned to private (secret) Facebook groups (invitation only, group and content not visible to anyone but participants) of varying sizes tailored to readiness to quit smoking [14,15]. Upon completion of the baseline assessment, participants were individually randomized to an incentive condition based on the following criteria: (1) those in the “personal” incentive condition were told they would receive a US $50 gift card if they commented daily on all 90 posts to their secret Facebook group, (2) those in the “altruistic” incentive group were told they would be given a US $50 gift card to the Just Give website to donate to a charitable organization of their choice if they commented on all 90 posts, (3) those in the no incentive condition were not given an incentive to comment. Upon completion of the intervention, data from secret groups were extracted from Facebook through the Facebook application programming interface (API) [29] for analysis.

### Intervention

All participants were invited to a secret Facebook group tailored to their stage of change: Precontemplation (ie, Not Ready to Quit); Contemplation (ie, Thinking About Quitting); or Preparation (ie, Getting Ready to Quit). Research staff made one daily Facebook post for 90 days tailored to their readiness to quit to each group. Posts were adapted from US Clinical Practice Guidelines [26] and Transtheoretical Model skills for smoking cessation [30]. Within each stage of change, TTM content included posts related to Decisional Balance or the 10 processes of change, according to TTM theory. For example, posts focusing on Decisional Balance were used in two of the three groups (Precontemplation and Contemplation) but focused more on eliciting the pros of change in Precontemplation and eliciting both pros and cons of change and reducing cons in the Contemplation groups, according to TTM theory. Table 2 gives an overview of the TTM content used in the different stages of change.

**Table 2:** Descriptive statistics of Transtheoretical Model (TTM) posts.

TTM content	Total number of posts	Used in stage^a^	Average number of comments	SD	Range
Decisional Balance	48	PC, C	3.00	3.00	0-13
Counter-Conditioning	27	C, P	0.74	1.77	0-9
Consciousness Raising	137	PC, C, P	1.21	1.79	0-11
Dramatic Relief	60	PC, C	0.68	1.30	0-6
Environmental Reevaluation	19	PC, C	2.00	2.29	0-8
Helping Relationships	18	C, P	1.22	1.66	0-6
Reinforcement Management	50	C, P	0.46	0.68	0-3
Stimulus Control	44	PC, C, P	0.54	0.81	0-4
Self-Liberation	30	C, P	0.90	1.64	0-7
Social Liberation	57	PC, C	1.79	2.70	0-16
Self-Reevaluation	22	PC, C, P	1.05	1.59	0-7

^a^C: Contemplation; P: Preparation; PC: Precontemplation.

Posts had a mix of imagery, text, and Facebook poll formats. Sample posts can be found in Multimedia Appendix 1. Post order was randomized, but the same order was used for all groups within each stage of change. When posts clearly referenced previous posts (eg, the post of the day before), these were randomized as blocks.

### Participants

Participants were 18-25 years old, English literate, and reported having smoked at least 100 cigarettes in their lifetime, currently smoked at least 3 days per week, and used Facebook at least 4 days per week.

### Measures

#### Engagement

Engagement was operationalized as the number of comments a Facebook post received (regardless of the number of individuals commenting). Comments were used as a primary measure of engagement instead of likes or posts because participants were instructed to comment on study posts every day.

#### Participant Baseline Characteristics

At baseline, demographic information included age, gender, race/ethnicity, education, and household income. We also assessed average number of days smoking per week (from which we computed percent smoking 7 days per week as “daily”) and presence of past year quit attempt (y/n) [31]. Time to first cigarette upon waking (<30 min or >30 min) was used as a measure of dependence [32]. Smoking goal was assessed with one item with seven response options, categorized into three categories: No goal, controlled or reduced smoking, and abstinence [33]. The Tobacco Smoking Stages of Change Questionnaire [15] assessed motivation to quit at baseline, categorizing smokers into one of three pre-action stages of change. Upon completion of the baseline assessment, participants were individually randomized to three incentive conditions: (1) personal incentive, (2) altruistic incentive, and (3) no incentive (see Procedure section).

#### Transtheoretical Model Post Content

Posts were classified according the TTM Decisional Balance or one of ten processes of change (see Table 1).

#### Post Features

Features of Facebook posts included group membership (one of seven Facebook groups), time of day (1 hour intervals), and day of the week each post was uploaded onto Facebook.

### Statistical Analysis

The relationship between engagement (total number of comments across all groups) and participant baseline characteristics and incentive group were examined using *t* tests. These analyses were based on total engagement throughout the intervention (718 total comments on posts based on TTM as well as other posts). To analyze our main research question of which TTM posts generated the best engagement, only the subset of posts that were based on TTM decisional balance and processes of change were selected (630 comments) and engagement was regressed on TTM post content. Since intervention posts were tailored to readiness to quit, these analyses were conducted separately by baseline readiness to quit smoking (Precontemplation, Contemplation, Preparation). In order to adequately address the over-dispersed outcome variable number of comments, negative binomial regression analyses were used [34], since fit indices suggested a better fit of negative binomial models compared to Poisson models. In the analyses for the Contemplation and Preparation stages, these models were additionally adjusted for excess zeros [35], which improved the model fit compared to negative binomial models without zero-inflation. Zero-inflated models estimate two equations simultaneously, one for the count model and one for the excess zeros. These excess zeros were regressed on the running number of the intervention day the individual post was made to the Facebook group (1-90; compared to posts with at least one comment). The rationale behind this was that as participants progressively disengaged with the Facebook intervention over time, a post made at a later time did not have the same likelihood to elicit engagement, as it had a lower chance to be read in the first place. The predictor TTM post content was dummy coded, and effect/deviation coding was used to estimate the difference in engagement between each individual theory post and the overall/average engagement (the mean of the outcome variable for a given level of the predictor was compared to the mean of the outcome variable for all levels of the predictor variable). The following covariates related to Facebook posts were examined: Group membership (one of seven Facebook groups), time of day (1-hour intervals), and day of the week each post was uploaded onto Facebook. These covariates were analyzed using analyses of variance (ANOVAs), and only significant variables were included in the final regression models. All analyses were conducted using Stata 11.2 [36].

## Results

### Sample Description

Of the 586 respondents who met criteria to participate, 230 signed online consent, and 79 completed a baseline assessment and were assigned to one of seven Facebook groups (number of participants mean 13, SD 5, range 7-22). Participants had a mean age of 21 (SD 2), 20% (16/79) were female, 80% (63/79) non-Hispanic white, and 18% (14/79) non-heterosexual. The median household income was between US $21,000 and $40,000, and 28% (22/79) reported a household income >US $60,000. Well over half (48/79, 61%) reported at least some college education while 56% (44/79) were employed. Of all participants, 75% (59/79) smoked daily and for an average of 3 years (SD 1). Mean age of initiation was 14 (SD 3) with regular smoking by age 16 years (SD 3), on average. The sample averaged 12 cigarettes/day (SD 8), 52% (41/79) smoked within 30 minutes of waking, and 57% (45/79) had made a past year quit attempt. With regard to their smoking goals, 30% (24/79) reported no goal, 60% (47/79) reported a reduction goal, and 10% (8/79) reported an abstinence goal. Of the 79 participants at baseline, 33 participants (42%) were in the Precontemplation stages of change, 36 were in Contemplation (46%), and only a minority of 10 (13%) were in Preparation. Last, 47% (37/79) of participants received no incentive for commenting daily, 28% (22/79) received an altruistic incentive, and 25% (20/79) received a personal incentive. Incentive conditions were equally distributed across stages of change (Precontemplation: 27% altruistic, 24% personal; Contemplation: 28% altruistic, 25% personal; Preparation: 30% altruistic, 30% personal).

The entire sample of 79 participants made a total of 718 individual comments to any intervention content. Of all 79 participants, 48 (60.8%) commented at least once and contributed an average of 15 comments per participant. Further, 42 (53.2%) of participants commented more than once. The 21 users with the most comments (26.6% of the entire sample) accounted for 593 comments (82.6% of all comments). No participant baseline characteristic significantly predicted intervention engagement (results not shown).

### Intervention Predictors of Engagement With Transtheoretical Model Posts

**Figure 1:**
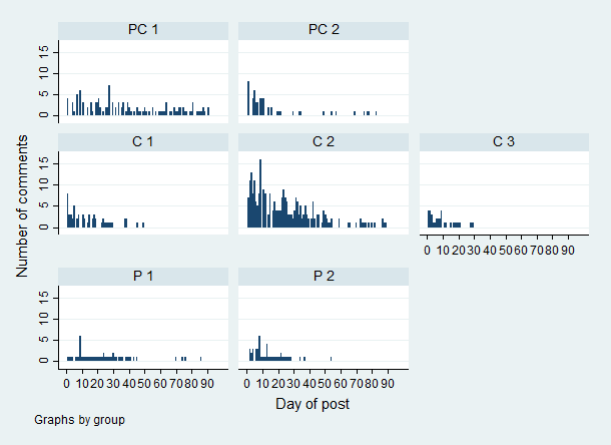
Engagement with Transtheoretical Model (TTM) posts in each of the 7 groups over time (C: Contemplation; P: Preparation; PC: Precontemplation).

In order to analyze which TTM content generated the highest engagement, only posts based on TTM theory were selected for analysis. This resulted in the selection of 512 posts, which generated 630 comments. Content was posted between 8 am and 6 pm, and a majority of posts (73%) were made between 1 pm and 3 pm (all Pacific Time). Timing of the post (time of day and weekday) was not significantly associated with engagement. Time of day reached marginal significance (time of day: *F*_9,502_=1.9, *P*=.05; weekday: *F*_6,505_=0.7, *P*=.67). Table 2 displays descriptive statistics according to the TTM-based posts. Of all 512 posts, 268 (52.3%) received at least one comment. The 125 posts with the most comments (24.4% of all posts) received 487 comments (77.3% of all comments). As can be seen in Figure 1, intervention engagement with TTM posts decreased over time in each of the seven Facebook groups (range of correlation coefficients from -.81 to -.55; all *P*<.001) and the number of comments varied by group (*F*_6,505_=29.7; *P*<.001). Thus, a dummy variable for Facebook group was the only covariate we subsequently included in all further analyses.

### Intervention Engagement According to Transtheoretical Model Post Content

The results of our negative binomial regression analyses to investigate which post content generated the best engagement are displayed in Table 3. Among participants in Precontemplation, Decisional Balance–based posts generated above average engagement compared to other posts (*P*=.01). Among those in Contemplation, Decisional Balance–based posts were also associated with above average engagement (*P*<.001) while Dramatic Relief and Self-Liberation posts were associated with below average engagement (*P*=.01 and *P*=.005). Last, among participants in Preparation, Consciousness Raising posts resulted in above average engagement (*P*=.009) and Helping Relationships–based posts were marginally but nonsignificantly associated with above average engagement (*P*=.08). Engagement differed between the two groups in Precontemplation (*P*<.001) and between the three groups in Contemplation (*P*<.001 and *P*=.02). Figure 1 shows engagement with TTM posts in each of the seven groups over time.

**Table 3:** Results of negative binomial regression analyses of Transtheoretical Model (TTM) content predicting intervention engagement, separately conducted for each baseline stage of change (adjusted for excess zeros in Contemplation and Preparation).

			b	Standard error	*t*	*P*
**Precontemplation**						
	**TTM content**					
		Consciousness Raising	Omitted^a^			
		Decisional Balance	0.685	0.275	2.5	.01
		Dramatic Relief	-0.581	0.956	-0.6	.54
		Environmental Reevaluation	0.285	0.383	0.7	.46
		Stimulus Control	-0.204	0.704	-0.3	.77
		Social Liberation	0.009	0.275	0.0	.97
		Self-Reevaluation	0.113	0.746	0.2	.88
		Group PC2^b^dummy	-0.883	0.190	-4.7	<.001
**Contemplation**						
	**TTM content**					
		Counter-Conditioning	Omitted^a^			
		Consciousness Raising	0.023	0.178	0.1	.90
		Decisional Balance	0.910	0.195	4.7	<.001
		Dramatic Relief	-0.526	0.211	-2.5	.01
		Environmental Reevaluation	0.237	0.262	0.9	.37
		Helping Relationships	0.127	0.477	0.3	.79
		Reinforcement Management	-0.374	0.558	-0.7	.50
		Stimulus Control	-0.276	0.414	-0.7	.51
		Self-Liberation	-0.669	0.239	-2.8	.005
		Social Liberation	0.330	0.202	1.6	.10
		Self-Reevaluation	-0.385	0.285	-1.4	.18
		Group C2^c^dummy	1.340	0.200	6.7	<.001
		Group C3^d^dummy	-0.595	0.258	-2.3	.02
		Inflated zeros **-** Intervention day	0.059	0.009	6.3	<.001
**Preparation**						
	**TTM content**					
		Counter-Conditioning	Omitted^a^			
		Consciousness Raising	0.628	0.239	2.6	.009
		Helping Relationships	0.521	0.293	1.8	.08
		Reinforcement Management	-0.118	0.283	-0.4	.68
		Stimulus Control	-0.152	0.307	-0.5	.62
		Self-Liberation	-0.569	0.868	-0.7	.51
		Self-Reevaluation	-0.258	0.553	-0.5	.64
		Group P2^e^dummy	0.031	0.213	0.1	.89
		Inflated zeros - Intervention day	0.105	0.020	5.3	<.001

^a^Dummy for first TTM content (Consciousness Raising for Precontemplation, Counter-Conditioning for Contemplation and Preparation) omitted to estimate each model.

^b^PC2: Precontemplation 2.

^c^C2: Contemplation 2.

^d^C3: Contemplation 3.

^e^P2: Preparation 2.

## Discussion

### Principal Findings

We investigated which intervention content based on the Transtheoretical Model generated the best engagement in a Facebook smoking cessation intervention for young adults tailored to participants’ readiness to quit smoking. Different intervention content generated varying levels of engagement according to TTM stage of change.

Participants in Precontemplation and Contemplation showed more than average engagement when posts were based on Decisional Balance. For those in Precontemplation, strategies focused more on increasing the pros of quitting, while posts in Contemplation groups acknowledged cons of quitting while simultaneously focusing on challenging the cons and increasing pros. Decisional Balance strategies are recommended by US Clinical Practice Guidelines for Smoking Cessation [26] and are consistent with motivational interviewing techniques of acknowledging ambivalence and guiding clients to focus on the positive aspects of quitting, effective with young smokers unmotivated to quit [37-39]. Changes in Decisional Balance have been shown to be associated with transitions from Precontemplation to Contemplation or Preparation for smoking [40-42] and other health risk behaviors including exercise [43] in previous studies. As applied to social media, Decisional Balance strategies can harness either a small social network (eg, an intervention group) or one’s larger Facebook social network to get help in generating pros and cons of smoking and smoking cessation.

For participants in Contemplation, we found that posts utilizing Dramatic Relief and Self-Liberation generated below-average engagement. Dramatic relief focuses on eliciting negative emotional responses to old behaviors (ie, smoking) and positive emotional responses to newly adopted behaviors (ie, quitting smoking). For Contemplation groups, Dramatic Relief posts were primarily focused on eliciting negative emotions related to smoking through, for example, links to the Legacy Foundation Truth Campaign YouTube videos of current or former tobacco users with severe consequences [44]. Findings suggest that posts focused on associating positive emotions with quitting or posts not linking to third-party websites (ie, YouTube) may have been a more effective strategy in implementing the Dramatic Relief process of change through social media. Self-Liberation posts focused on asking participants to take small steps toward quitting and to share their experience with other group members. It is not possible to know whether lower than average engagement suggests “steps” were not taken or were just not reported in groups. Future posts using Self-Liberation strategies could focus on having participants share strategies in extremely incremental steps or eliciting ideas from participants or group members about which steps they would be willing to take or would suggest for their peers to elicit more sharing in groups.

Posts based on Consciousness Raising resulted in above average engagement among participants in Preparation. The TTM posits that Consciousness Raising takes a less active role than processes such as Counter-Conditioning, Stimulus Control, and Reinforcement Management to help move people from Preparation to Action stages of change. Nevertheless, participants were most engaged with these posts, suggesting that those ready to quit are interested in information about quitting smoking. Posts focused on the health benefits of quitting, for example, may be most engaging in Preparation groups.

Engagement diminished over time in all groups, which is consistent with previous studies on health behavior change interventions using social media [23-25]. However, we also found that engagement was independent of time of day and day of week, suggesting that a Facebook smoking cessation intervention can deliver critical information to participants and has the potential to get them to engage every day. This is especially promising since 70% of Facebook users report daily use [11]. Furthermore, more than 50% of participants in the present intervention actively engaged two times or more, showing that the intervention was interesting enough for them to come back more than once. Compared to online smoking cessation interventions not using social media [10], about the same proportion of participants engaged with the intervention at all (63% vs 61% in our study). However, the participants in our study engaging at least once commented an average of 15 times, which compares favorably to an average of 1.4 visits made to a smoking cessation website [10]. Consistent with previous research [12], these findings suggest that social media can be harnessed to engage participants in smoking cessation and other health behavior change interventions.

We did not find baseline participant characteristics that predicted differences in engagement; however, participants clearly showed varying intensities of engagement. This suggests that we either did not capture important baseline predictors of engagement or that substantial variance in engagement gets introduced at the group level. Indeed, intervention engagement did differ by group. Unfortunately, this feasibility study with seven groups lacked the adequate sample size to investigate group-level factors such as group size, group composition (eg, gender ratio, percentage of daily smokers, or future smoking intentions), or group processes (eg, do a couple of very active participants motivate others to be more active as well?) that may have caused these group level differences. Future studies with larger samples are needed to investigate this topic.

Previous studies of both behavioral smoking cessation counseling [26] and Web-based smoking cessation intervention [27] suggested that higher engagement may lead to better smoking cessation outcomes. Of our 79 participants, 60 (75.9%) completed the follow-up assessment at end of treatment (3-month follow-up). Of these, 7 participants (11.7%) reported 7-day point prevalence abstinence at end of treatment. Participants with self-reported abstinence made an average of 22.4 (SD 21.9) comments, compared to 8.3 (SD 15.1) comments among non-abstinent participants. This difference was borderline statistically significant in a Wilcoxon-Mann-Whitney test (*z*=1.9; *P*=.05), indicating that engagement was associated with more favorable smoking cessation outcomes in our intervention. When conservatively assuming those not followed-up were still smoking (intent-to-treat or ITT), this difference was statistically significant: abstinent: 22.4 comments (SD 21.9); non-abstinent: 7.8 comments (SD 13.9); *z*=2.0; *P*=.04. However, it should be noted that due to our small participant sample, the achieved test power to detect this effect was only 56% (61% respectively for ITT analysis). A trial adequately powered to investigate this research question more in depth is warranted.

### Limitations

Our findings should be interpreted with several limitations in mind. This study relied on a self-selected convenience sample of young adult smokers using Facebook, and the sample was predominantly male and white. Social networks were formed as part of the intervention rather than derived organically based on the participants’ Facebook or other preexisting social network. Strategies are needed to recruit more female and ethnic minority participants through Facebook targeting (eg, placing ads in locations where more ethnic minority smokers reside) and using images to target women and non-white smokers. Further, our intervention was tailored to baseline readiness to quit smoking in accordance with the TTM, and thus participants in each stage of change received different intervention content. This study design feature was accounted for by examining engagement stratified by baseline readiness to change; however, it precluded us from comparing engagement with TTM content between different stages of change. Our analysis of post features was theory-guided and focused on the TTM. We thus did not investigate how other aspects of Facebook posts, such as sentiment (positive/negative) or semantic content were related to participant engagement [45]. This should be examined in future studies. Comments are a conservative measure of engagement; indeed, the absence of commenting does not necessarily mean a person did not see or make some cognitive or behavioral change as a result of an intervention post. These, however, were impossible to measure in the context of this study conducted entirely on Facebook. In addition, we were not able to take the quality and depth of engagement (eg, comment content, length) into account. However, overall we argue that comments to Facebook posts are likely a more meaningful measure of active intervention engagement [46,47], compared to previous studies that looked at number of website visits or time spent viewing specific websites as measures of engagement (eg, [10,20]). In addition, time of day that the intervention content was posted was not randomized, and the majority of content was posted between 1 pm and 3 pm Pacific Time.

### Conclusions

Social media such as Facebook provide unprecedented opportunities to reach large numbers of young adult smokers with smoking cessation interventions. However, it is crucial to investigate and improve participant engagement in these types of interventions. This study has important implications for interventions with young adult smokers on social media. Results underscore the importance of tailoring intervention content to readiness to quit smoking to maximally engage young adults in social media interventions. Decisional Balance was most engaging to those not ready to quit (Precontemplation, Contemplation stages of change), and Consciousness Raising was most engaging to those in Preparation. Results suggest that in order to increase participant engagement, social media smoking cessation interventions should use posts that increase the pros of quitting for participants in Precontemplation, posts that increase the pros while challenging the cons for participants in Contemplation, and posts that provide information on quitting smoking for participants in Preparation. Although social media are generally integrated into the lives of young adults, strategies are still needed to improve participant engagement in social media smoking cessation intervention over time. Future studies should also examine how engagement with specific intervention content is related to treatment outcomes.

## Appendix

Multimedia Appendix 1 Sample Facebook posts in precontemplation, contemplation, and preparation.

## Abbreviations

C: contemplation
ITT: intention to treat
P: preparation
PC: precontemplation
TTM: Transtheoretical Model
