# Supplementary material for: Smoking Cessation Intervention on Facebook: Which Content Generates the Best Engagement?
Source: J Med Internet Res. 2015 Nov 11;17(11):e244. doi: 10.2196/jmir.4575 (PMC4704894; doi:10.2196/jmir.4575)

Multimedia Appendix 1. Sample Facebook posts in Precontemplation, Contemplation, and Preparation

PRECONTEMPLATION

Decisional Balance

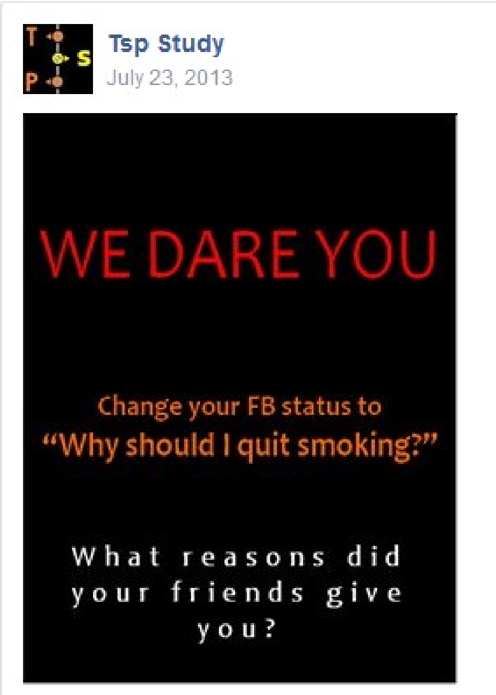

Consciousness Raising

Tsp Study
October 21, 2013

What was your reason for picking up that first cigarette? Was it to be cool and independent, to experiment, to look older, something else?

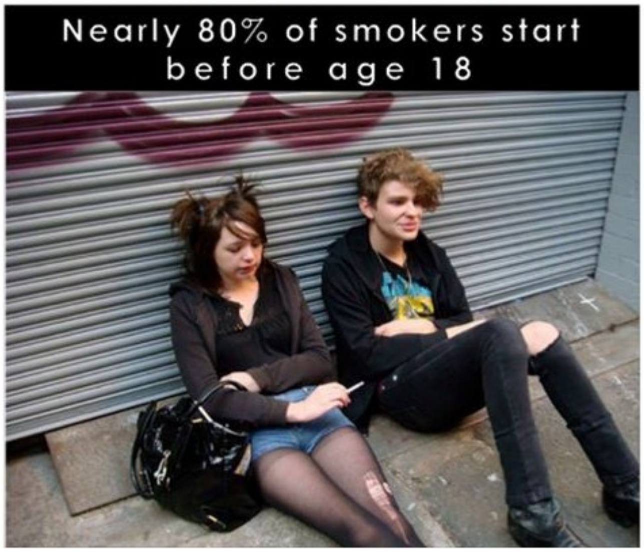

Social liberation

Tsp Study
September 10, 2013

Check this link to see what the Tobacco Control Laws are like in your state. Be sure to know the smoking legislation if you plan on traveling. Post to the group and tell us what you think about the laws in your state.
http://www.rwjf.org/.../interactive-tobacco-map-provides-late...

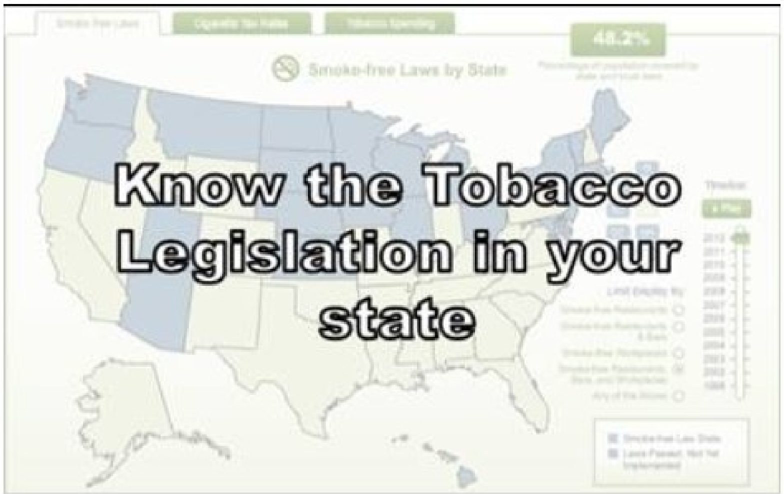

Environmental Reevaluation

Tsp Study
August 31, 2013

Cats are exposed to second hand smoke toxins through breathing in the smoke around them, AND when they groom themselves. How can we help Fifi to stop smoking?

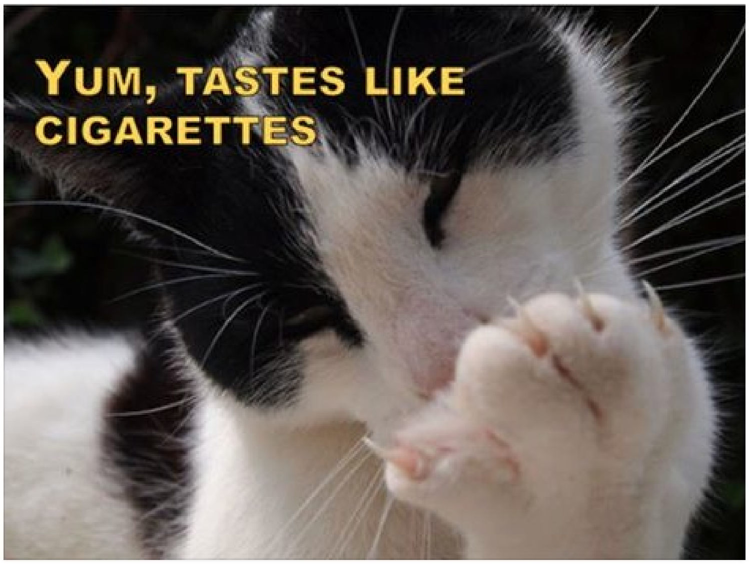

CONTEMPLATION

Decisional Balance

Tsp Study
July 17, 2013

Now that you've made your list of Cons for quitting smoking, let's consider your top reason NOT to quit. Post your #1 Con for smoking here to the group and tell us why it's your biggest concern. Finish this sentence: "I want to quit smoking, but I'm worried that ________", then comment on other group members' concerns about quitting.

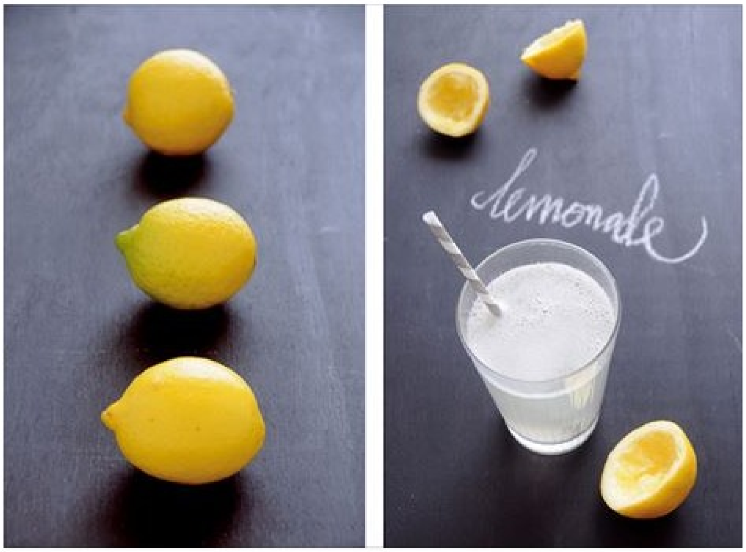

Self-Liberation

Tsp Study
August 30, 2013

Turn your small steps towards quitting up a notch. Tell someone special outside of your FB lives about your small step or goal. Share with the group who that special person is and how things went when you told them:

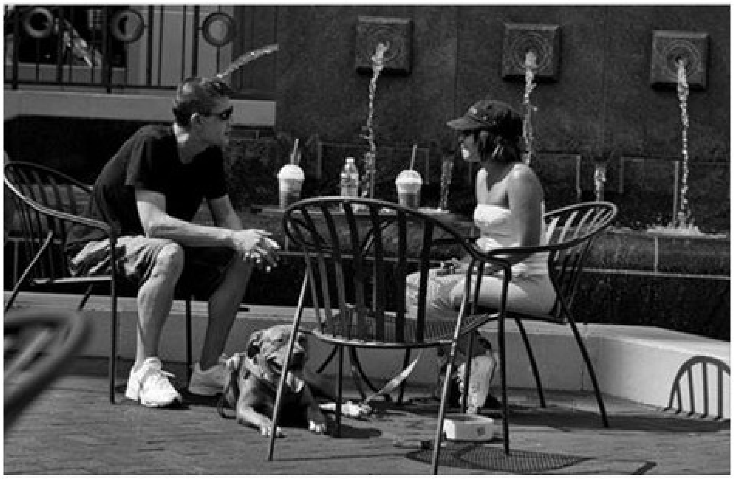

Helping Relationships

Tsp Study
August 21, 2013

Who's that one friend you can count on? The person who will listen to you about what's going on, always willing to help? Post&Share who you will be able to trust as your supporter when you decide to quit and how they will support you:

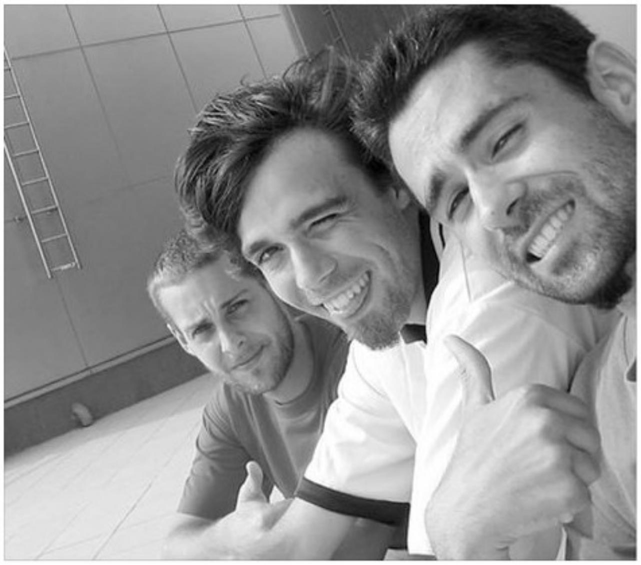

Dramatic Relief

Tsp Study
September 14, 2013

Click here to see how one Tobacco company called young adult smokers "replacement smokers" on record. What does it feel like to be part of a big, expensive marketing strategy? http://www.thetruth.com/facts/replacement-smokers/

1. VOLUME

Younger adult smokers are the only source of replacement smokers. Repeated government studies (Appendix B) have shown that:

- Less than one-third of smokers (31%) start after age 18.
- Only 5% of smokers start after age 24.

Thus, today's younger adult smoking behavior will largely determine the trend of Industry volume over the next several decades. If younger adults turn away from smoking, the Industry must decline, just as a population which does not give birth will eventually dwindle. In such an environment, a positive RJR sales trend would require disproportionate share

PREPARATION

Self-Reevaluation

Tsp Study
August 14, 2013

When you're a nonsmoker, what will be different about your day to day? Tell us what you're looking forward to

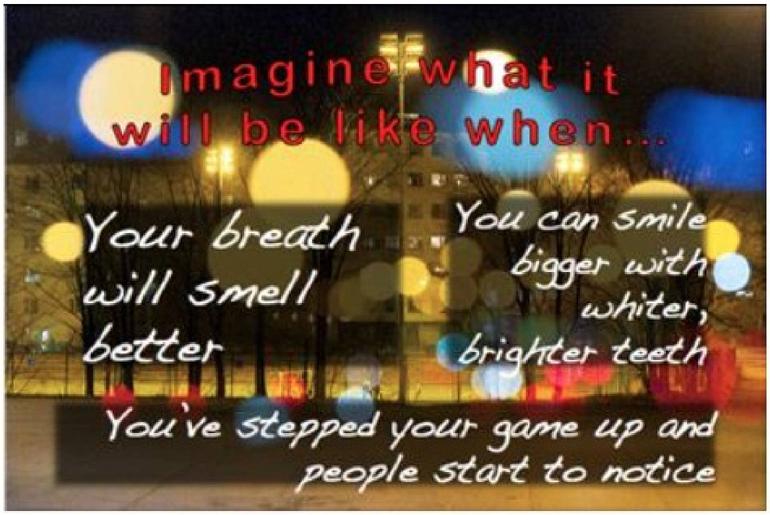

Counter-Conditioning

Tsp Study
October 16, 2013

One of the best ways to combat a nicotine craving is to keep your hands busy! How can you plan to keep your hands occupied when you get hit with a sudden urge to smoke?

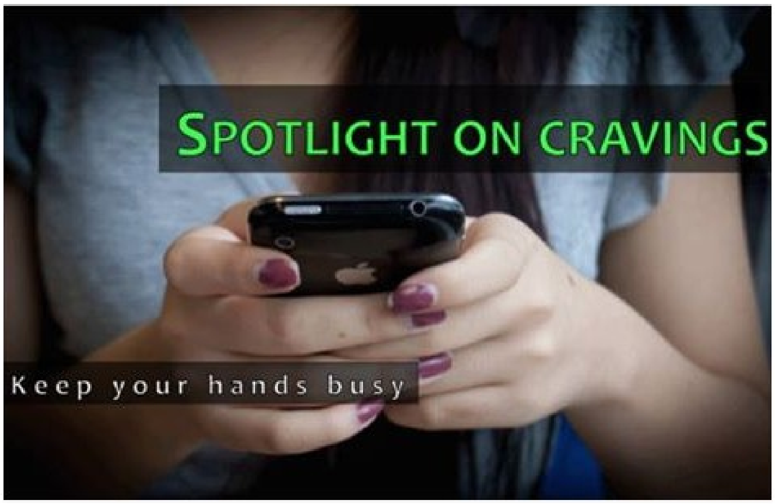

Stimulus Control

Tsp Study
September 12, 2013

To prepare for your Quit Date, think about how you will handle mornings without cigarettes? Post your answers to our questions here and keep them handy for that morning when you might need them!

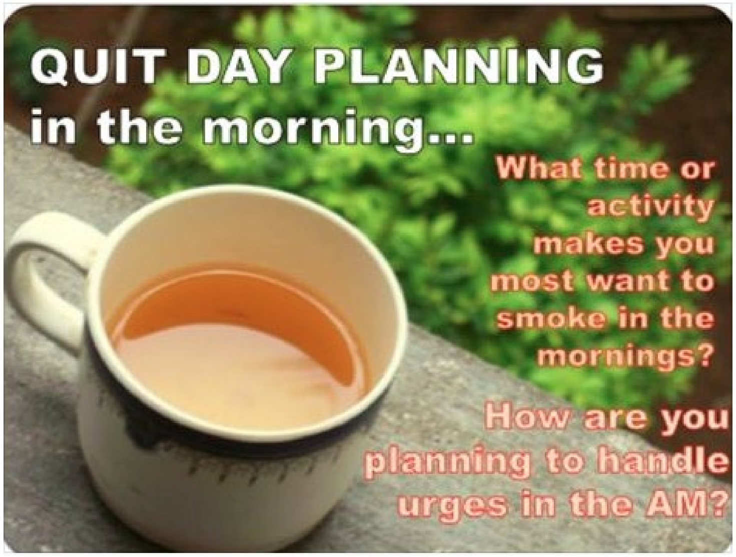

Reinforcement Management

Tsp Study
September 30, 2013

How are you focusing on what you're gaining by quitting? Take a pic of your daily reminders and post them here! Anyone got a savings jar?

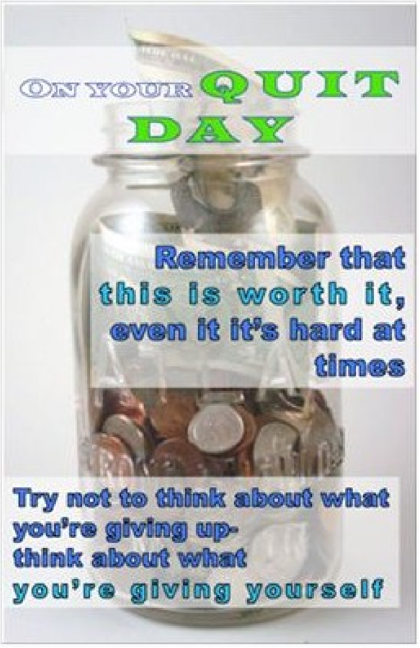

Supplement: Multimedia Appendix 1 [file jmir_v17i11e244_app1.pdf]
